# Sexual functioning as predictor of depressive symptoms and life satisfaction in females with Polycystic Ovary Syndrome (PCOS)

**DOI:** 10.12669/pjms.36.7.2562

**Published:** 2020

**Authors:** Muneeba Shakil, Farzana Ashraf, Amina Wajid

**Affiliations:** 1 Muneeba Shakil, Department of Humanities, COMSATS University Islamabad, Lahore Campus, Pakistan; 2 Dr. Farzana Ashraf, Department of Humanities, COMSATS University Islamabad, Lahore Campus, Pakistan; 3 Amina Wajid, Department of Humanities, COMSATS University Islamabad, Lahore Campus, Pakistan

**Keywords:** polycystic ovarian syndrome, depressive symptoms, sexual functioning, life satisfaction

## Abstract

**Background & Objective:**

PCOS is the one of the frequently diagnosed endocrine syndrome in Pakistani women. However, little attention has been devoted to empirical investigation of the role of sexual dysfunction in developing depressive symptoms and reducing life satisfaction. Our objective was to determine the contribution of Sexual dysfunction in developing depressive symptoms and life dissatisfaction in females diagnosed with PCOS.

**Methods:**

This correlation study was carried out from 1^st^ September 2019 to 15^th^ February 2020 at COMSATS University Islamabad, Lahore Campus. A sample of 60 women with PCOS between reproductive age 18 – 38 years (M*age* = 27.86, SD = 4.58) was selected through purposive sampling technique from the government hospitals of Lahore, Pakistan. The participants responded to a Demographic Information Form, Female Sexual Functioning Index, Siddiqui Shah Depression and Life Satisfaction Scale.

**Results:**

Correlation analysis revealed significant positive link of sexual dysfunction with depressive symptoms and negative link with life satisfaction level. Further, regression analysis revealed sexual functioning as significant predictor of depressive symptoms only.

**Conclusion:**

Sexual dysfunction play a major role in general mental health of females, therefore, screening and identification as well as preventive measure need to be introduced at early level of development. In addition, current study findings suggest that once females are diagnosed with PCOS, they should be provided sufficient counselling services in order to deal with depressive symptoms and feeling of low life satisfaction. Further, targeted interventions and counselling services may also facilitate in optimising patient care.

## INTRODUCTION

Polycystic ovary syndrome (PCOS) is a common hormonal disorder in women of reproductive age with demonstrations of excessive production of male hormones (i.e., androgen), prolonged or infrequent menstrual periods which results in overall sexual dysfunction. Sexual functioning is a bio-psycho-social phenomenon which is highly affected in case of presence of PCOS.^1^ While looking into general manifestation of PCOS, it was noticed that women diagnosed with PCOS have polycystic ovaries mostly developed due to follicles (i.e. a number of small collections of fluid) which can result in enlarged ovaries, reducing their ability to release eggs, leading to fertility issues.^2^ It is the most common female hormonal disorder with 52% prevalence in South Asian women. Specifically, in Pakistani women, the rate is much higher as compared to white population.^3^ Although, this phenomenon is medically accepted and treated well in other parts of the world, yet in Pakistan it represents a different picture. Being part of collectivist culture, married women are expected to have children without acceptance of any medical complication which induces in them depressive symptoms in specific and decreases their satisfaction with life in general.

Assessment of hormonal variations in a sample of Pakistani women suffering from polycystic ovary syndrome revealed elevated body mass index, Luteinizing Hormone (LH), insulin, prolactin, androstenedione and testosterone which are also responsible for sexual desire and performance.^4^,^5^ Though, the link of gynaecological issues such as PCOS with sexual dysfunction is often bidirectional, it is difficult to ascertain which had appeared first.^6^ Elevated androgen levels due to PCOS bring changes in body that effects sexual functioning. Whereas, increased testosterone level effect the sexual response cycle including motivation, desire and response to sexual intercourse. The exact association of androgen levels and sexual functioning is still understudied as few studies report its negative effect on sexual functioning.^7^ As the perceived body image of women with PCOS is deteriorated with a perceived loss of feminine identity, therefore, the discrepancy between societal standards of ideal body and the actual outward appearance of these women creates a negative impact on their thinking patterns, attitude and overall quality of life.^1^

The aforementioned literature includes studies of PCOS women with sexual dysfunction, depression, and quality of life. However, there is gap in knowledge with respect to the predictive relationship of sexual dysfunction with depressive symptoms and life satisfaction in women diagnosed with PCOS. This phenomenon is under-studied in Pakistan with reference to multiple aspects, and empirical investigation is needed to shift focus of researchers to this health-related issue. The present study was carried out with the objective to determine the role of sexual dysfunction in predicting depressive symptoms and life dissatisfaction in Pakistani women with PCOS. It was hypothesized that sexual dysfunction may be a positive predictor of depressive symptoms and negative predictor of life dissatisfaction in women with PCOS.

## METHODS

This correlation study was carried out from 1^st^ September 2019 to 15^th^ February 2020 at COMSATS University Islamabad, Lahore Campus of Pakistan to determine the predictive relationship of sexual dysfunction with depressive symptoms and life satisfaction of women with PCOS. A sample of 60 women between reproductive age 18 – 45 years (M*age* = 27.86, SD ± 4.58) was selected through purposive sampling from different government hospitals of Lahore, Pakistan. Sample size was estimated by using RaoSoft software of sample size calculation with significance of 5% (2 tailed) and 95% confidence interval. To control the confounding effects, only those women were taken as study participants who were diagnosed by a gynaecologist. The criteria used for their diagnosis was first clinical assessment, then abdominal ultrasound for determination of polycystic ovaries and lastly a hormonal assay if required; moreover, certain inclusion/ exclusion criteria were also developed. Participants were included in the study if they were: (1) in the reproductive age group; (2) married; (3) had been diagnosed with PCOS by a gynaecologist for the last six months; (4) were undertaking treatment; (5) were living with their husbands (6) did not had children; (7) permanent resident of Pakistan and (8) can read and write Urdu. They were excluded from participation in the study if they: (1) suffered from a mental illness; and (2) had any other physical illness or disability.

The participants were administered a demographic sheet, The Siddiqui Shah Depression Scale (SSDS), Female Sexual Function Index (FSFI) and Life Satisfaction Sub-Scale (LSSS). All measures were available in URDU language. SSDS contains 36 items assessing mild, moderate and severe levels of depressive symptoms.^8^ FFSI is 19 items self-reporting measure for sexual functioning scores ranging from 2 to 36. High scores are indicator of healthy sexual functioning.^9^ LSS is the five items Subscale of Subjective Wellbeing Scale used in the current study to assess the life satisfaction of women with PCOS.^10^

Initially, current study was approved by ethical review board of COMSATS University, Lahore with number Ref.No.CUI/LHR/HUM/075 on August 15, 2019. Followed by that, informed consent was sought from the women participants. Later, all self-report measures were administered. All the participants were assured of the privacy and confidentiality of the provided information. IBM SPSS Statistics package 24 was used to carry out the analysis where Pearson product moment correlation and Regression analyses were used to determine the predictive relationship of sexual dysfunction with depressive symptoms and life satisfaction of women with PCOS.

## RESULTS

A sample of 60 women (diagnosed with PCOS) of reproductive age (from 18 to 38 *M* = 27.86, *SD*+4.58 years). Among these, 5% (n=3) did matriculation, 47% (n=30) Master and 48% (n=29) M. Phil/ MS. Sixty two percent (n=37) were living in joint family. In addition, 33% (n=20) of participants were with working status and 67% (n=40) were house wives. Three percent of the women had monthly income of less than 10,000 (n=2), 14% (n=8) between 11,000 to 25,000, 25% (n=15) between 26,000 to 50,000, 30% (n=18) between 51,000 to 100,000 and 28% (n=17) more than 1000,000. Pearson product moment correlation analysis revealed (Table-I) significant negative correlation of sexual functioning with depressive symptom (*p<.01*) and significant positive relationship with life satisfaction (*p<.01*). Table-II illustrated that sexual functioning is negative predictor of depressive symptoms (*p<.01*) but insignificant in case of life satisfaction (p.>01) while controlling the demographic variables (i.e., age, education, work status and monthly income) (Table-II).

**Table-I:** Relationship between sexual dysfunction, depressive symptoms and life satisfaction of women with PCOS.

Measure	Depressive Symptoms	Life satisfaction
Sexual Functioning Index	-.278**	.266**

*Note:* *. Correlation is significant at the 0.01 level (2-tailed).

**Table-II:** Regression Analyses Predicting depressive symptoms and life dissatisfaction from sexual dysfunction in women with PCOS (N=60).

Predictors	Depressive symptoms	Life satisfaction
Model 1	Β	β
1- Age	-.231	.229
2- Education	.063	-.085
3- Family system	-.112	.084
4- Work status	.161	-.214
R^2^	.110	.123
∆R^2^	.050	.061
F	1.754	1.93
Model 2		
1- Age	-.085	.639
2- Education	.002	-.227
3- Family system	-.211	1.241
4- Work status	.156	-1.566
5- Sexual functioning	-.306*	1.881
R^2^	.18	.177
∆R^2^	.11	.101
Incremental R	.06	.041
F	2.37*	2.32*

*Note:* **p<.05, ***p<.001, Work Status; working=1, non-working=2,

## DISCUSSION

In the current study, predictive role of sexual functioning in determining depressive symptoms and life satisfaction was assessed. Results revealed that sexual functioning is inversely related to depressive symptoms and positively related to life satisfaction. However, sexual functioning was only found to be a significant negative predictor of depressive symptoms in women with PCOS. One of the most stressful factors in Indian subcontinent women’s life is infertility. Past literature from Pakistan suggests that infertility effects the satisfaction and quality of life in women with PCOS.^11^,^12^ In our study all the participants were infertile which could justify their sexual dysfunction and its association with depressive symptoms and life satisfaction. In a recent investigation Pakistani women with PCOS exhibited health risks including hypertension, diabetes, infertility, miscarriages and hyperandrogenism along with low quality of life with depression as a significant contributor.^13^

Women also suffer psychological distress in terms of depression, anxiety, low self-esteem and low quality of life as causal factors of sexual dysfunction and infertility.^14^,^15^ This might relate to the predictive role of sexual functioning in depressive symptoms of women with PCOS from this region as well. Sexual functioning is said to be significantly related to anxiety, stress and depression.^16^,^17^ In women of reproductive age depression is said to be the strongest predictor of female sexual functioning. An inverse relationship between depression and all the subscales of sexual functioning index was found with sexual pain as predictor of depression.^18^ Findings of our study suggest that sexual functioning is a strong predictor of depressive symptoms in women with PCOS. As sexual functioning is affected by a number of factors in this syndrome, women from this study reported to experience pain while having sexual intercourse. This pain might be a strong factor contributing to sexual dysfunction and its role as a predictor of depressive symptoms in our sample. PCOS women report their partners to be less sexually active and attracted to them. Moreover, they also reported to have low level of sexual desire and thoughts.^19^ This lack of satisfaction with their sex life and lack of communication on part of their husbands might be contributing to develop depression as a result of having this endocrine syndrome.

**Fig.1:**
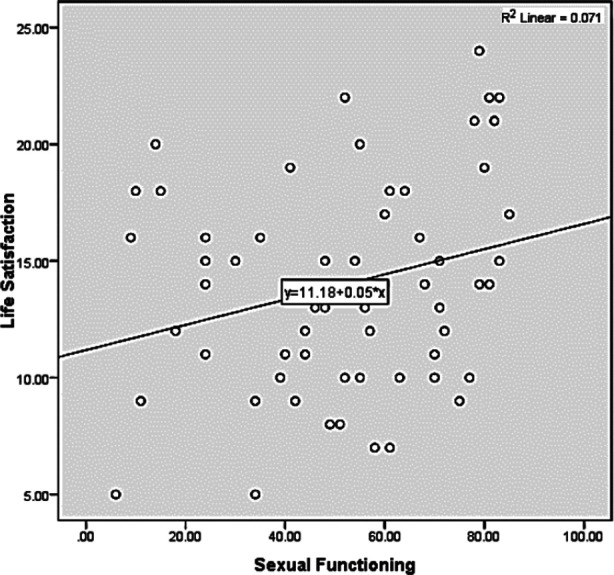
Relationship between sexual functioning and life satisfaction.

**Fig.2:**
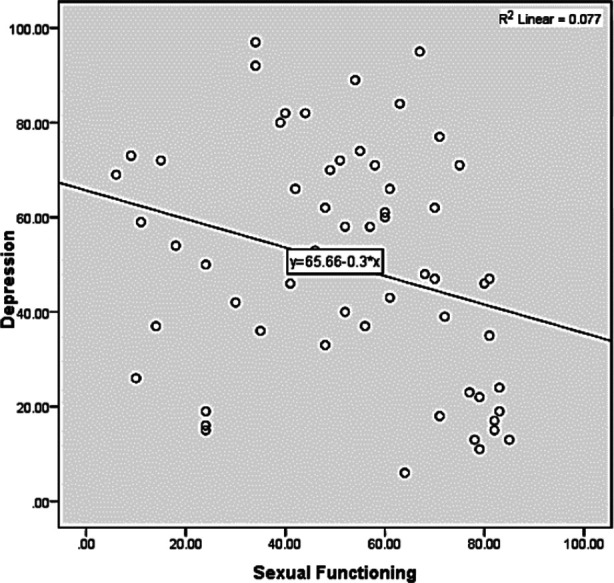
Relationship between sexual functioning and depressive symptoms.

According to a past finding, 70% to 80% of women suffering from PCOS in Indian subcontinent have infertility, however, women from this region shy away discussing their sexual life and problems related to it. If these women do not get pregnant within stipulated time their sexual desire is often reduced.^20^ Constant pressure from society for bearing a child has a negative effect on the cognition of women as they are often blamed for the infertility in the couple. Out of this pressure sexual intercourse is performed as a duty and perceived as a burden contributing to negative thoughts and appraisal affecting sexual response leading to depressive symptoms.

Thus, this study adds to the medical literature by filling gap in knowledge with respect to the role of sexual dysfunction and its contribution in developing depressive symptoms in women diagnosed with PCOS. The clinical significance of this study lies in applying the knowledge while taking sexual history from the patient to identify the problem areas and plan effective interventions. The patients and their partners are curious and need to know all the facts related to their medical problem from their doctor.

### Limitations of the study

The recruitment of our study population was one of the biggest limitations as majority of sample was collected from government hospitals of Lahore where many of the women with PCOS were consulting private hospitals due to lack of sufficient facilities and care in government hospitals. Further, the hospital administration itself did not cooperate with the researchers for data collection. There could also be a limitation to extend our results to fertile PCOS patients who have less contact with the health care system. Our restricted inclusion criteria and the aforementioned limitations left us with a small sample size. Furthermore, due to time and financial restrains a comparison sample was also not included in the study; therefore, it is recommended to in cooperate a comparison sample along with a longitudinal study carried out to determine sexual and clinical changes over time.

## CONCLUSION

Literature suggest that depression is most prevalent in women with PCOS and certain symptoms of the disease are associated with depression including quality of life. However, no literature is available on the predictive role of sexual functioning in the relationship between depression and satisfaction with life.^21^ The findings of our study indicate that sexual functioning is a negative predictor of depressive symptoms in women suffering from PCOS; however, sexual functioning did not predict lower satisfaction with life in these women.

### Recommendation

It is recommended that affected women could be referred for a consultation with psychologist or sexologist who could improve their quality of life with simple interventions. As future endeavour prospective clinical studies are suggested to evaluate possible targeted treatments in order to regain normal sexual function in PCOS patients.

### Authors’ Contribution:

**MS:** Conceived, designed, manuscript writing is responsible for the accuracy and integrity of the work. **FA:** Did data analysis, editing of manuscript and final review.

**AW:** Did data collection and reviewed literature.
